# Deep Spatio-Temporal Graph Network with Self-Optimization for Air Quality Prediction

**DOI:** 10.3390/e25020247

**Published:** 2023-01-30

**Authors:** Xue-Bo Jin, Zhong-Yao Wang, Jian-Lei Kong, Yu-Ting Bai, Ting-Li Su, Hui-Jun Ma, Prasun Chakrabarti

**Affiliations:** 1 Artificial Intelligence College, Beijing Technology and Business University, Beijing 100048, China; 2 China Light Industry Key Laboratory of Industrial Internet and Big Data, Beijing Technology and Business University, Beijing 100048, China; 3 Department of Computer Science and Engineering, ITM SLS Baroda University, Vadodara 391510, India

**Keywords:** time series data prediction, spatio-temporal network, self-optimization, GRU, graph neural network

## Abstract

The environment and development are major issues of general concern. After much suffering from the harm of environmental pollution, human beings began to pay attention to environmental protection and started to carry out pollutant prediction research. A large number of air pollutant predictions have tried to predict pollutants by revealing their evolution patterns, emphasizing the fitting analysis of time series but ignoring the spatial transmission effect of adjacent areas, leading to low prediction accuracy. To solve this problem, we propose a time series prediction network with the self-optimization ability of a spatio-temporal graph neural network (BGGRU) to mine the changing pattern of the time series and the spatial propagation effect. The proposed network includes spatial and temporal modules. The spatial module uses a graph sampling and aggregation network (GraphSAGE) in order to extract the spatial information of the data. The temporal module uses a Bayesian graph gated recurrent unit (BGraphGRU), which applies a graph network to the gated recurrent unit (GRU) so as to fit the data’s temporal information. In addition, this study used Bayesian optimization to solve the problem of the model’s inaccuracy caused by inappropriate hyperparameters of the model. The high accuracy of the proposed method was verified by the actual PM2.5 data of Beijing, China, which provided an effective method for predicting the PM2.5 concentration.

## 1. Introduction

In recent years, along with the development of the economy, people’s living standards have been improving daily, and people are paying more attention to their happiness. Yet, air quality is sure to directly affect people’s happiness. Therefore, time series prediction technology has been widely used in air quality prediction. Among the many factors affecting air quality, PM2.5 is among the most significant. PM2.5 refers to particles with an aerodynamic equivalent diameter of less than or equal to 2.5 microns in the ambient air. It can remain suspended in the air for a long time. The higher its concentration in the air, the more serious the air pollution is. PM2.5 comes from various sources, mainly from human activities, such as coal combustion, urban dust, automobile exhaust emissions, and industrial pollution sources. Although the size of PM2.5 is tiny, it is a component with little content in the Earth’s atmospheric composition. Still, compared with other atmospheric particles that are coarser and more abundant, PM2.5 has a large area coverage, intense activity, a long staying time in the atmosphere, and a long transportation distance. It easily carries toxic and harmful substances (heavy metals, microorganisms, etc.) and can change the precipitation and temperature modes. Thus, it has a more significant impact on human health and atmospheric environmental quality [1]. Therefore, it is of great significance in accurately predicting the concentration of PM2.5.

With the increasing impact of air pollution on people, air pollution has become a topic of concern, and the prediction of pollution has also become an essential means of controlling air pollution. With the development of science and technology, obtaining PM2.5 concentration and other air pollutant data is convenient. By analyzing and studying past PM2.5 concentrations data, researchers can obtain the changing patterns of the PM2.5 concentration to a certain extent. However, because of the very complex formation mechanism and changing process of PM2.5 concentration, together with the effect of the temporal dimension (time series) and spatial dimension (geological distance), its data are a kind of non-stationary time series with complex nonlinear and noise characteristics, which increases the difficulty of predicting the PM2.5 concentration. Therefore, establishing an air quality prediction model with high accuracy and real-time performance that can fully consider space–time nonlinearity has always been a hotspot in the time series data prediction field.

Researchers have explored air pollution prediction from different research directions and established a variety of prediction methods. In the 1960s and 1970s, some developed countries first began to study methods of predicting air pollution, of which the most important is the potential prediction [2]. This is mainly based on the diffusion of pollutants, but a potential prediction cannot reveal the quantitative pollutant concentration. In order to obtain better prediction results, statistical methods and machine learning technology have been widely used in air prediction modeling. The statistical method involves computer-built statistical probability models based on data and uses the models to analyze and predict. Simple statistical methods mainly use the autoregression model (AR) [3] and autoregressive integrated moving average model (ARIMA) [4] to predict air quality. Machine learning technology uses a computer algorithm that can be automatically improved by experience. Machine learning technology that is applied to air quality prediction includes artificial neural networks (ANNs) [5], support vector regression (SVR) [6], random forest (RF) [7], etc. Although the above method is easy to implement for predicting air quality, and the model is relatively simple and can be aptly explained, it is difficult to establish a model of nonlinear data, and it is also difficult to effectively express highly complex data. Therefore, it cannot handle large datasets with high complexity and strong nonlinearity for actual PM2.5 prediction.

In addition to algorithms based on traditional statistical and machine learning technologies, more and more research has begun to use deep learning technology to build prediction models. Relying on its powerful modeling ability and nonlinear processing ability, the deep learning method has been widely used in air quality prediction. Standard deep learning network models include the recurrent neural network (RNN) [8], gated recurrent unit (GRU) [9], long short-term memory network (LSTM) [10], temporal convolution network (TCN) [11], etc. These networks and their variants have been widely used in air quality prediction and have achieved good prediction accuracy. However, most of the above deep learning methods emphasized the fitting analysis of time series but neglected or simplified the impact of spatial transmission between different regions, thus decreasing the prediction accuracy.

Compared with the above methods, the graph neural network (GNN) is a framework that uses deep learning technology to learn graph-structured data directly. Its excellent performance has greatly attracted scholars’ attention and in-depth exploration. The commonly used graph neural networks include a graph convolution network (GCN) [12], graph attention network (GAT) [13], graph sampling and aggregation network (GraphSAGE) [14], etc. These networks and their variants have been successfully applied to air quality prediction. Because of their strong ability to fit nonlinear chart-structured data, they show high accuracy and robustness. However, these networks often consider only the spatial dimension and simultaneously fail to consider the time and space dependence. In order to solve this problem, many researchers began to combine recurrent neural networks and graph neural networks to form graph recurrent neural networks (GRNs) [15]. A GRN usually converts graph data into sequences, recursively evolving and changing during training. This combination can establish a full model based on the dependence of the two dimensions of space and time and can also effectively solve the common problems of gradient disappearance/explosion in time series prediction, thus improving the prediction accuracy.

Although the graph recurrent neural network has shown its effectiveness in many fields because of its excellent nonlinear fitting ability and powerful spatio-temporal information capture ability, just like other deep learning methods, its performance may also be seriously affected by hyperparameters selected on the basis of empirical knowledge or multiple attempts. This operation requires too much experimental time and computational resources, and the obtained hyperparameters may not be optimal, as they will lead to the unstable performance and limited accuracy of the model [16]. Therefore, applying an effective strategy to find the most optimal hyperparameter set of the model is the critical step in solving the above problems.

To sum up, if air quality prediction only focuses on the information in a single time or space dimension while ignoring or simplifying the fitting of time series and the analysis of common spatial transmission effects between different locations, the prediction accuracy will decrease. Therefore, based on the ability of graph neural networks to capture spatial information, as well as the advantages of a recurrent neural network to effectively solve the common problems of gradient disappearance/explosion in time series prediction, this paper proposes a new time series prediction network with the self-optimization ability of spatio-temporal graph gated recurrent units called BGGRU. The main contributions of this paper are as follows:

(1) BGraphSAGE for the spatial dimension: A GNN model (GraphSAGE) is used to extract the spatial features, and the Bayesian method is used to optimize the model’s hyperparameters. The model can extract hidden spatial information while eliminating the unstable performance of the model caused by the experience-based selection of hyperparameters.
(2) BGraphGRU for the temporal dimension: Graph convolution is used to replace the linear operation of the GRU with the Bayesian hyperparameter optimization method so that the network can extract the temporal dependency with suitable hyperparameters.
(3) BGGRU with the combined model: This consists of a spatial dimension (BGraphSAGE) module and temporal dimension (BGraphGRU) module, which can thoroughly learn the data from the time and space dimensions so as to effectively improve the prediction accuracy and generalization performance of the prediction model.

The rest of this paper is organized in the following way: Section 2 introduces the related work in this field; Section 3 presents the method proposed in this study in detail; and Section 4 describes the setup and results of the experiment, in which the proposed combined network model was used to predict PM2.5 in Beijing, China. Section 5 is the conclusion of this paper and suggests prospective work.

## 2. Related Work

### 2.1. The Traditional PM2.5 Prediction Method

Traditional PM2.5 prediction methods include the statistical method and machine learning technology method. Because of its relatively simple structure, the statistical method mainly considers the formation mechanism of PM2.5. It is widely used in air quality prediction. Liu et al. [17] combined ARIMA with numerical prediction to predict the daily and hourly PM2.5 concentration in Hong Kong. Zeng et al. [18] studied the relationship between PM2.5 and meteorological factors in Chengdu within 24 h. They used the generalized additive model to predict the concentration of PM2.5. The traditional prediction method based on statistics has limitations because the formation of PM2.5 is very complex.

Based on historical data, machine learning for air pollution prediction can model the nonlinearity of actual air pollution data, thus producing a higher prediction accuracy. Shahriar [19] et al. evaluated hybrid models consisting of ARIMA, ANN, SVM, PCR, DT, and Catboost. Among these models, Catboost had the best performance. The ARIMA-ANN and DT methods also provided acceptable results. Caroline et al. [20] constructed and evaluated an SVM to predict ground PM2.5 in densely populated cities with complex terrains. The final results proved the potential of SVM as a prediction model in other tropical cities. Rui et al. [21] used multi-layer perceptron (MLP) to analyze and predict environmental PM2.5 in eight core regional cities in China and found that reducing gaseous pollutants is crucial to regulating PM2.5.

With the explosion of data, statistical models and machine learning methods with simple structures can no longer provide the complex modeling capabilities required for big data, and they easily fall into overfitting.

### 2.2. PM2.5 Prediction Method Based on Deep Learning

In recent years, the deep learning method has attracted significant attention for air quality prediction because of its strong learning ability and ability to fit nonlinear data. Dongming Qin et al. [22] used a combined model consisting of two deep networks (CNN and LSTM). Among them, the CNN model was used to extract the features of input data, and the LSTM model was used to consider the time dependence of pollutants. The prediction results show that it improves the prediction performance compared with classic models. H. Kaimian et al. [23] used LSTM to predict the PM2.5 concentration in Tehran, and the prediction results could explain 80% of the PM2.5 variability. K. Nagrecha [24] et al. proposed a CNN network system to analyze past sensor measurements and predict air pollutant concentrations. The result was equivalent to the most advanced prediction system in this field and was usually superior to any advanced prediction system.

Although these networks have been widely applied in air quality prediction, most networks do not fully mine data characteristics from the two dimensions of time and space. Moreover, most deep learning models focus on Euclidean space, but the air quality monitoring station distribution often fails to match the ideal conditions of Euclidean space.

### 2.3. PM2.5 Prediction Method Based on Graph Neural Network

A graph neural network, referring to the algorithm, uses a neural network to learn, extract, and mine the features and patterns in graph-structured data to meet the needs of graph learning tasks such as clustering, classification, prediction, segmentation, generation, etc. A graph is composed of nodes and edges, usually denoted as G=(V,E), where $V=\{V_{1},V_{2}\ldots V_{n}\}$ represents the node set, and $E=\{E_{1},E_{2}\ldots E_{m}\}$ represents the edge set. Graph neural networks can adapt to complex structures a priori, such as by defining the relationship between multiple concepts, describing complex nonlinear structures, etc.

In addition, compared with other neural network models, graph neural networks can model the overall characteristics of data from two aspects, i.e., structure and function. Therefore, a graph neural network has greater universality in spatio-temporal data modeling and information mining.

In recent years, graph neural networks have been widely used in air quality prediction on account of the above characteristics. P. Zhao [25] et al. proposed a method using multi-attention spatio-temporal graph networks (MASTGN) to predict the air pollutant concentration in China and Japan. Shuo Wang et al. [26] proposed a graph-based model called PM2.5-GNN, which can capture long-term dependencies. In order to combine the two advantages of strong interpretability and feature extraction ability, Hz et al. [27] combined the PM2.5 dispersion partial differential equation with a deep learning method based on the newly proposed DPGN model and used PM2.5 monitoring data obtained every hour. Zhao [28] proposed a comprehensive prediction method to extract the spatial propagation between adjacent places. The methods mentioned above try to mine information in the spatial dimension rather than solve the problems of gradient disappearance/explosion (common issues in time series prediction), which will reduce the prediction accuracy. Therefore, they fail to combine the advantages of graph neural networks in capturing spatial information with the recurrent neural network in processing time series.

To sum up, traditional prediction methods and those based on deep learning have difficulty mining the data features in the space and time dimensions. In recent years, graph neural networks have provided a new idea for air quality prediction due to their ability to mine spatial characteristics. In this paper, we propose a new time series prediction network with the self-optimization ability of spatio-temporal graph gated recurrent units, which combines three elements: the spatial information extraction ability of a graph neural network, the advantages of a recurrent neural network that can effectively solve the gradient problem in time series prediction, and the powerful hyperparameter optimization ability of the Bayesian method.

## 3. Methods

### 3.1. Overall Structure of BGGRU

**Figure 1:**
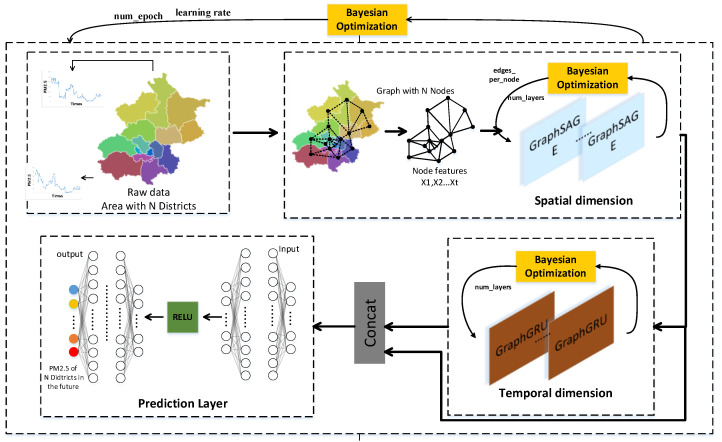
The overall structure of BGGRU.

**Figure 2:**
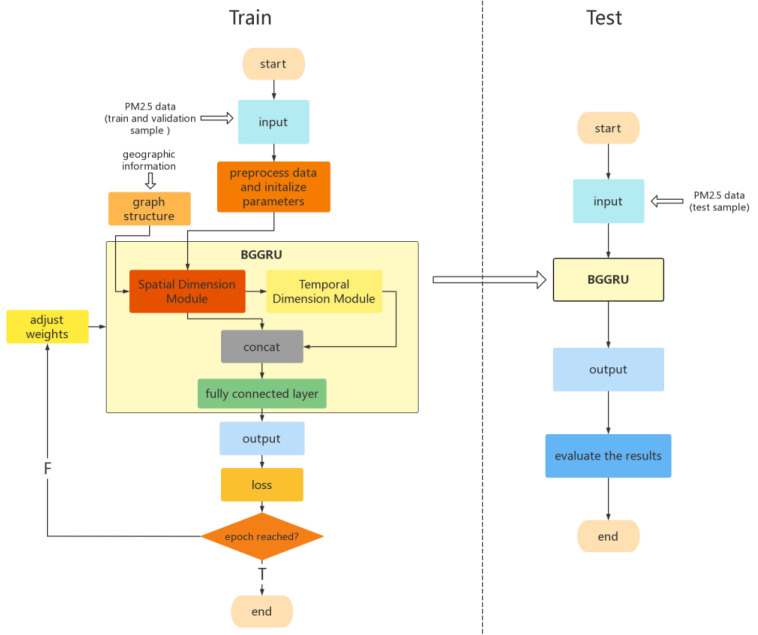
The learning diagram of BGGRU.

BGGRU is composed of two modules: the spatial dimension module (BGraphSAGE) and the temporal dimension module (BGraphGRU). They jointly exploit the spatio-temporal relationship in the data. As shown in Figure 1, the first part of BGGRU is the spatial dimension module, using BGraphSAGE to aggregate local features, generate embedding for each node in the graph, and extract the spatial features from the data. The second part of BGGRU is the temporal dimension module, using BGraphGRU to jointly model spatio-temporal dependencies while maintaining the spatial characteristics of the input. Lastly, the future concentration of PM2.5 is predicted through two fully connected layers. The model also uses the Bayesian method to optimize the hyperparameters of each module in the model and the model as a whole. Additionally, the learning diagram for BGGRU is shown in Figure 2, which indicates the learning process of the model. In the training stage, we first preprocess the training and validation datasets (see details in Section 4.1), randomly initialize the network parameters, and generate the graph adjacency matrix describing the graph structure through known geographic information. Then, the BGGRU network proposed in this paper is used to extract the spatio-temporal dimension features from the data and predict the future concentration of PM2.5. Finally, the loss function (see details in Section 4.2) is used to calculate the prediction error, and the network weights are adjusted later. The above steps are repeated until the number of epochs is reached. In the test stage, the test dataset is sent to the trained BGGRU, and the future concentration of PM2.5 is predicted through the network. The performance of the network is evaluated by the evaluation function (see details in Section 4.2).

In addition, the model also uses a skip connection for the two modules of space and time. In deep neural networks, when the layer is deep, the model might often succumb to overfitting and gradient disappearance/explosion problems, resulting in the failure to update the parameters in the shallow layer. A skip connection means the next layer, including not only the information of the current layer but also the newly obtained information of the current layer after nonlinear transformation. This method solves the problems of overfitting and gradient disappearance/explosion of the model to a certain extent.

As shown in Figure 1 and Figure 2, the proposed model in this paper adds a skip connection mechanism. That is, the final fully connected layer receives not only the output of BGraphGRU but also the output of the first BGraphSAGE. The skip connection can accelerate the stability of the model and prevent overfitting.

#### 3.1.1. Spatial Dimension Module of BGGRU (BGraphSAGE)

**Figure 3:**
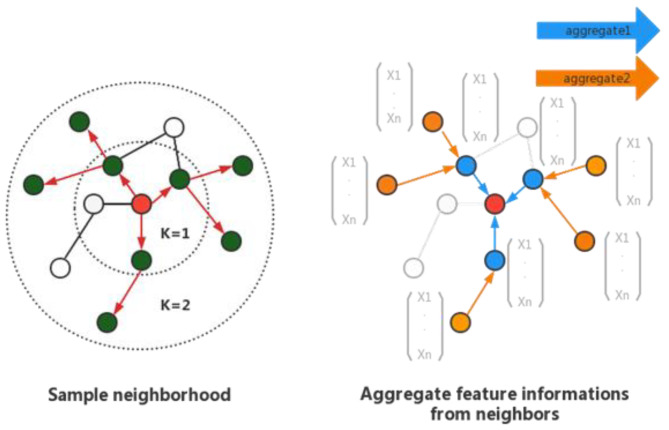
Visual illustration of the GraphSAGE sample and aggregate approach.

Traditional graph embedding algorithms (based on matrix decomposition and random walk) need to use the information of all nodes in the iterative process to learn the vector representation. These previous approaches are inherently transductive. GraphSAGE [29] includes sampling and aggregation. First, the connection information between nodes is used to sample their neighbors, and then the information of adjacent nodes is continuously fused through multi-layer aggregation functions. The structure of GraphSAGE is shown in Figure 3. The method proposed in this paper also uses GraphSAGE to aggregate the features of local nodes, generate embedding for each node in the graph, and extract the spatial features from the data.

The graph is represented as ς=(υ,ε), where υ is the set of nodes, and ε is the set of edges. In each iteration K, each node will aggregate the embeddings of all adjacent nodes by taking the average of these vectors. Then, the aggregated vector is concatenated to the current embedding vector, and the output is calculated through a linear layer with a sigmoid activation function.

We also used the Bayesian method to automatically optimize the hyperparameters of the spatial dimension module (see Section 3.2 for details).

#### 3.1.2. Temporal Dimension Module of BGGRU (BGraphGRU)

The GRU is a variant of LSTM, which is also proposed to solve gradient problems in long-term memory and backpropagation. In general, the GRU, like LSTM, is mainly used for temporal feature extraction from time series data, and its actual performance is similar to that of LSTM in many cases, but its calculation is simpler and easier to implement compared to LSTM. Therefore, the BGraphGRU proposed here has the same chain structure as GRU. Still, a graph convolution operation (GraphSAGE) is used to replace these linear transformations and extract the structural features of snapshots at each time. After that, BGraphGRU is used to solve the problem of long-term dependence and effectively learn the temporal characteristics of input graphs. In this way, the spatio-temporal correlation can be jointly modeled while maintaining the spatial structure of the input. BGraphGRU’s formula is shown below.

The GRU introduces the concepts of the reset gate and update gate, thus modifying the calculation method of hidden states in the recurrent neural network. The BGraphGRU proposed in this paper has the same chain structure as GRU but uses a graph convolution operation (GraphSAGE) to replace the original linear transformation. The function of the reset gate’s action is the same as that of the GRU, and the subject is the hidden state in front. Additionally, the function is to determine how much past information needs to be forgotten. The formula for the reset gate is as follows:

(1) $$r_{t}=\sigma (W_{r}A_{t}+GraphSAGE_{r}^{k}(h_{t-1},{\hat{A}}_{t-1})+b_{r})$$

where $A_{t}\in R^{N\times N}$ is the input of BGraphGRU at time *t*, $h_{t-1}\in R^{N\times d}$ is the hidden state at time *t* − 1, and $W_{r}\in R^{N\times d}$ and $b_{r}\in R^{d}$ are the weight matrix and bias matrix of the reset gate.

In fact, the update gate can be understood as a combination of the forget gate and input gate in LSTM. The subject of its function is the hidden unit of the current time and the previous time, and its function is to decide how much useful information needs to be transferred down at the current time and previous time. The formula for the update gate is as follows:

(2) $$z_{t}=\sigma (W_{z}A_{t}+GraphSAGE_{z}^{k}(h_{t-1},{\hat{A}}_{t-1})+b_{z})$$

where $A_{t}\in R^{N\times N}$ is the input of GraphGRU at time *t*, $h_{t-1}\in R^{N\times d}$ is the hidden state at time *t* − 1, and $W_{z}\in R^{N\times d}$ and $b_{z}\in R^{d}$ are the weight matrix and bias matrix of the update gate.

Next, BGraphGRU will calculate the candidate hidden state to assist in the later hidden state calculation. The formula is as follows:

(3) $${\tilde{h}}_{t}=\tanh(W_{hA}\cdot A_{t}+W_{hh}(r_{t}*GraphSAGE_{h}^{k}(h_{t-1}))+b_{h})$$

It can be seen from the above formula that the reset gate controls how the hidden state of the previous time step flows to the candidate hidden state of the current time step. The hidden state of the last time step may contain all of the historical information of the time series up to the last time step. Therefore, the reset gate can discard historical information irrelevant to the prediction.

Finally, the calculation of the hidden state $h_{t}$ of time step *t* uses the update gate $z_{t}$ of the current time step to combine the hidden state $h_{t-1}$ of the previous time step with the candidate hidden state ${\tilde{h}}_{t}$ of the current time step. The formula is as follows:

(4) $$h_{t}=(1-2t)*GraphSAGE_{h}^{k}(h_{t-1})+z_{t}*{\tilde{h}}_{t}$$

We also used the Bayesian method to automatically optimize the hyperparameters of the temporal dimension module (see Section 3.2 for details).

### 3.2. Bayesian Hyperparameter Optimization

While the graph neural network is widely used in air quality prediction, the selection of the model’s hyperparameters, like in other deep learning methods, often depends on experience; not only does this consume lots of time and resources, but the obtained hyperparameters are not necessarily optimal.

Therefore, the critical step in solving the above problems is to use an effective strategy to find out the optimal hyperparameter set of the model. In recent years, Bayesian optimization has become widely used in solving black box function problems and has become the mainstream for hyperparameter optimization. Bayesian optimization is a global one. The objective function only needs to meet local smoothness assumptions, such as uniform continuity or Lipschitz continuity. The introduction of the acquisition function for effective exploration and utilization can obtain the approximate solution of complex objective functions with shorter evaluation times. This is why we optimized the model’s hyperparameters through the Bayesian method to ensure the prediction model’s performance [30]. The formulas are as follows.

The first step in the Bayesian method is to assume a functional relationship between the loss function to be optimized and the hyperparameter of the model:

(5) $$p^{*}=\underset{p\in P}{\arg\min loss(p)}$$

In this formula, $p^{*}$ is the optimal combination of hyperparameters obtained from the Bayesian hyperparameter optimization algorithm, P is the set of all hyperparameters, p is the set of input hyperparameters, and loss(•) is the objective function needed to be optimized. In our model, the hyperparameters to be optimized included the number of training epochs (num_epoch), the learning rate of the overall model (learning rate), the number of BGraphSAGE layers (num_layers of BGraphSAGE), the number of edges of each node (edges_per_node) in the graph, and the number of BGraphGRU layers (num_layers of BGraphGRU). The mean absolute error defines the loss function, and the formula is as follows:

(6) $$loss(p_{j})=\frac{1}{n}\sum_{j=1}^{n}\left|{\hat{y}}_{i}(p_{j})-y_{i}\right|$$

In this formula, $p_{j}$ is the *j*-th hyperparameter combination, y is the actual value, and $\hat{y}(p_{j})$ is the model output results using the hyperparameter combination $p_{j}$.

The next step of the Bayesian method is to construct a dataset $D=\{(x_{1,}y_{1}),(x_{2,}y_{2})\ldots (x_{i,}y_{i})\}$, in which $x_{i}$ is the *i*-th hyperparameter set, and $y_{i}$ is the error of the output result under that set of hyperparameters:

(7) $$y_{i}=loss(p_{i})$$

Next, the Bayesian method will use limited observation points to estimate the function distribution and obtain the surrogate model M, which obeys the Gaussian distribution *G* with variance k and mean μ. The posterior probability $p(y|x_{i},D)$ is derived from dataset *D*. The expression of the specific function M is gained from dataset *D*:

(8) p(loss)=G(loss;μ,k)

(9) $$p(loss|D)=G(loss;\mu_{loss|D},k_{loss|D})$$

The function used to determine the rules of the following observation point is called the acquisition function a(p). It will measure the impact of the observation point on the fitting of the surrogate model M and select the point with the most significant impact to perform the following observation.

(10) $$p^{*}=\arg\max a(P,p(y|x))$$

The ultimate goal of Bayesian hyperparameter optimization is to continuously optimize our estimation of the objective function loss(•) with the gradual increase in observation points under the influence of the acquisition function a(p) and, finally, estimate the minimum value of the objective function loss(•). Therefore, the above steps will be repeated until the optimal hyperparameters are selected or the maximum number of iterations is reached.

## 4. Experiment and Results

### 4.1. Dataset and Experimental Environment

**Figure 4:**
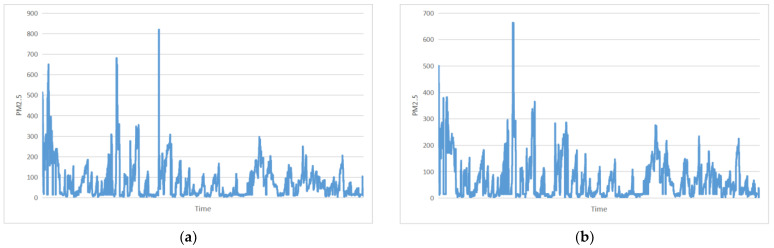
Examples of data from different districts in Beijing, China. (**a**) Haidian; (**b**) Xicheng.

This study used PM2.5 concentration data obtained from 16 districts in Beijing, China (Dongcheng District, Xicheng District, Chaoyang District, Fengtai District, Shijingshan District, Haidian District, Shunyi District, Tongzhou District, Daxing District, Fangshan District, Mentougou, Changping District, Pinggu District, Miyun District, Huairou District, and Yanqing District) from 1 January 2017, to 1 April 2017, as the target variable. The sampling time interval is 1 h, with a total of 2160 samples, in which the proportion of the training set, verification set, and test set is 8:1:1. Shown in Figure 4 are the data from Haidian District and Xicheng District. The data type in the other districts is about the same. The model takes 24 data points as an input sample to predict the 25th data point. The datasets are publicly available at https://github.com/btbuIntelliSense/Beijing-PM2.5-Concentration-Data (accessed on 14 January 2023).

This study conducted all experiments on a computer equipped with a 12th Gen Intel (R) core (TM) i5-12500h processor, 4800 mhz, and 16 GB memory. All models were built using PyTorch, an open-source deep learning framework.

Unfortunately, there happened to be some values missing in the actual dataset in this experiment. The method for dealing with missing values in this work was to replace the missing value at time *t* with the data at time *t* − 1. Should 24 consecutive data be missing (a whole day), the data from the previous day would have to be used in place of the missing data.

In this experiment, the nodes of the graph refer to the 16 districts in Beijing, China. The edges connecting the nodes are determined by the distance between the separate districts (the distance between them was calculated by the longitude and latitude of the districts). Each node is assigned to its *N* (the number of edges_per_node) nearest adjacent districts.

### 4.2. Loss Function and Evaluation Index

The loss function used in this experiment was the mean absolute scaled error (MASE), and the formula is as follows:

(11) $$loss=\frac{\left|\sum_{i=0}^{N-1}{\hat{y}}_{i}-y_{i}\right|}{\sum_{i=0}^{N-1}y_{i}}$$

In this formula, ${\hat{y}}_{i}$ represents the predicted value of PM2.5 obtained through the model, and $y_{i}$ represents the actual value of PM2.5.

In this experiment, four evaluation functions were used to evaluate the prediction performance of the model, that is, the root-mean-square error (RMSE), mean square error (MSE), mean absolute error (MAE), and coefficient of determination (*R*^2^). Their formulas are as follows:

(12) $$RMSE=\sqrt{\frac{1}{N}\sum_{i=1}^{N}{\left({\hat{y}}_{i}-y_{i}\right)}^{2}}$$

(13) $$MSE=\frac{1}{N}\sum_{i=1}^{N}{\left({\hat{y}}_{i}-y_{i}\right)}^{2}$$

(14) $$MAE=\frac{1}{N}\sum_{i=1}^{N}|{\left({\hat{y}}_{i}-y_{i}\right)}^{2}|$$

(15) $$R^{2}=1-\frac{\sum_{i}^{N}{\left({\hat{y}}_{i}-y_{i}\right)}^{2}}{\sum_{i}^{N}{\left({\bar{y}}_{i}-y_{i}\right)}^{2}}$$

Among them, N represents the total number of samples in the dataset, $y_{i}$ and $\bar{y}$ represent the actual value and the average actual value of PM2.5, and ${\hat{y}}_{i}$ represents the predicted value of the PM2.5 concentration obtained through the model. For RMSE, MSE, and MAE, the smaller the value, the better the model’s prediction performance. For *R*^2^, the closer the value is to 1, the better the model’s prediction performance.

### 4.3. Experimental Results

#### 4.3.1. Bayesian Hyperparameter Optimization Results

The Bayesian method was used to optimize the five hyperparameters: num_layers of BGraphSAGE, num_layers of BGraphGRU, num_epoch, learning rate, and edges_per_node. The results of Bayesian optimization are shown in Table 1. The following experiments used the optimal hyperparameters to predict the PM2.5 concentration.

#### 4.3.2. Comparative Prediction Results

Nine models were used as the baseline, namely, TCN [31], ConvLSTM [32], CNN-LSTM [33], GRU [34], BidirectionalLSTM (BiLSTM) [35], LSTM [36], BidirectionalGRU (BiGRU) [37], BayesLSTM (bLSTM) [38], and BayesGRU (bGRU) [39]. The dataset and data division ratio used in the baseline models were the same as those of the proposed model. We compared the average PM2.5 prediction results of the above baseline models and the proposed model for 16 districts in Beijing, China. The errors in the prediction results are shown in Table 2.

As shown in Table 2, BGGRU ranks number one in the comprehensive order, followed by bGRU [39] and bLSTM [38]. It can be seen that the model proposed in this paper is better than the control models for the four evaluation indicators used in this experiment. The value of *R*^2^ is very close to 1, and compared with the control models ranking second and third in the comprehensive indicators, RMSE, MSE, and MAE, respectively, increased by 61%, 84%, and 61% on average. As the baseline models focus more on information in the time dimension but ignore the air transmission effect between different locations, the model proposed in this paper first uses a graph neural network to extract the characteristics of the spatial dimension information and then extracts the time dimension information from the data through a model combining a graph neural network and a recurrent neural network, thus fully considering the characteristics of the data in the space–time dimension. Therefore, the proposed model has better prediction results than the other models in predicting air quality.

#### 4.3.3. Ablation Study

First, the prediction results of Beijing’s average PM2.5 using only the spatial dimension module of BGGRU were compared with the model proposed in this paper. The comparison results are shown in Table 3.

As shown in Table 3, the prediction results using the model proposed in this paper are better than those using the spatial dimension network alone. The RMSE, MSE, and MAE of the model proposed in this paper are 64%, 87%, and 71% higher than those using the spatial dimension module alone, and its *R*^2^ is also closer to 1. This shows that it is not enough to consider only the spatial dimension, but it is essential to consider the spatio-temporal dimension.

In addition, we also compared the impact of using Bayesian hyperparameter optimization with that of selecting hyperparameters through experience on the prediction results of Beijing’s average PM2.5. The results of the hyperparameter set obtained with Bayesian optimization and empirical selection are shown in Table 4, and the comparison results are shown in Table 5.

It can be seen from the above results that the model using Bayesian hyperparameter optimization is better than the model using empirical selection in terms of prediction results, which shows that Bayesian hyperparameter optimization can improve the prediction ability of the model to a certain extent. Thus, compared with the method of selecting hyperparameters through experience, using the Bayesian method to optimize hyperparameters can improve the model’s performance and save a lot of time and computing resources. Moreover, it enjoys a better mathematical theoretical basis and interpretability.

#### 4.3.4. Display and Analysis

**Figure 5:**
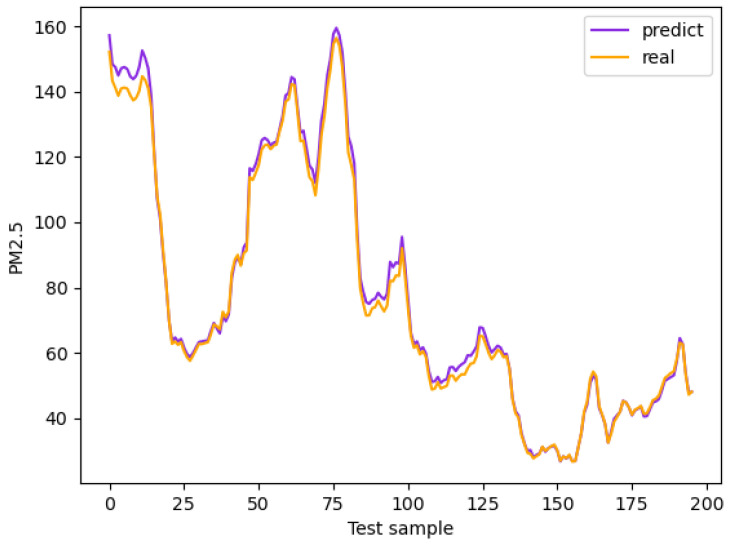
Average PM2.5 prediction results in Beijing, China.

**Figure 6:**
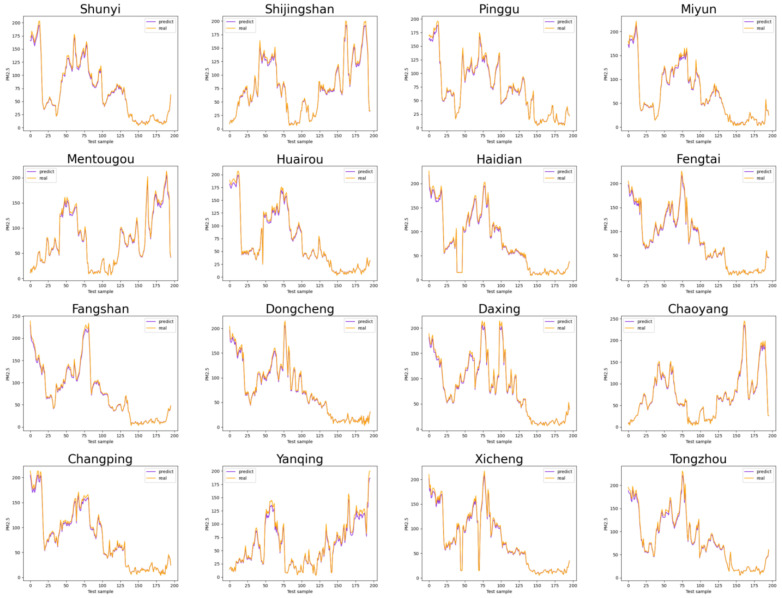
PM2.5 prediction results for 16 districts in Beijing, China.

This section shows the average and respective PM2.5 prediction results in Beijing using the model proposed in this paper with the above data, as shown in Figure 5 and Figure 6.

It can be seen from Figure 5 and Figure 6 that the prediction of the proposed model is consistent with the trend of the actual values, which reflects the proposed model’s effectiveness in PM2.5 prediction after extracting spatio-temporal information.

## 5. Conclusions and Future Work

Because changes in air quality data are affected by both the time dimension and spatial dimension, this paper proposes a deep spatio-temporal graph network with self-optimization to predict the concentration of PM2.5. In the combined network model, firstly, a graph neural network is used to extract the characteristics of the spatial dimension information from the data. Then, while maintaining the spatial structure of the data, a graph recurrent neural network is used to extract the time dimension information from the data. The model can fully extract and mine the characteristics of the data from the two dimensions of time and space and solve the common gradient problems in time series prediction to improve prediction accuracy. In addition, we also effectively solve the model’s inaccuracy caused by inappropriate hyperparameters by using Bayesian optimization to select the optimal hyperparameters of the model. The effectiveness of the proposed method was proven through an experiment on predicting PM2.5 in 16 districts of Beijing, China.

In future research, we will test our proposed model on a more complex time series dataset, and we will try to combine the graph neural network with different recurrent neural networks and their variants to further improve the overall performance of the prediction model.

## Figures and Tables

**Table 1:** Bayesian hyperparameter optimization results.

Hyperparameter	Hyperparameter Set with Bayesian Optimization
num_layers of GraphSAGE	4
num_layers of GraphGRU	1
num_epoch	11
learning rate	0.0327
edges_per_node	3

**Table 2:** Errors in prediction results of different models.

Model	RMSE	MSE	MAE	*R* ^2^
TCN [31]	26.91	724.33	21.65	0.4
ConvLSTM [32]	25.46	648.65	20.05	0.47
CNN-LSTM [33]	24.85	617.76	19.76	0.49
GRU [34]	15.93	253.85	11.49	0.86
BiLSTM [35]	15.78	248.88	11.93	0.87
LSTM [36]	15.27	233.35	10.66	0.87
BiGRU [37]	13.22	174.65	9.74	0.91
bLSTM [38]	7.02	49.28	5.15	0.97
bGRU [39]	6.71	45.09	5.07	0.97
BGGRU (proposed) *	**2.66**	**7.11**	**1.99**	**0.99**

* The bold part shows the best performance of different models of the same indicator.

**Table 3:** Comparison of prediction results between the spatial module alone and the overall model.

Model	RMSE	MSE	MAE	*R* ^2^
GGRU	7.39	54.75	6.88	0.96
**BGGRU**	**2.66**	**7.11**	**1.99**	**0.99**

The bold part shows the best performance of the same indicator.

**Table 4:** The results of the hyperparameter set obtained with Bayesian optimization and empirical selection.

Hyperparameter	Hyperparameter Set with Bayesian Optimization	Hyperparameter Set with Empirical Selection
num_layers of GraphSAGE	4	3
num_layers of GraphGRU	1	3
num_epoch	11	10
learning rate	0.0327	0.05
edges_per_node	3	3

**Table 5:** Comparison of prediction results between the hyperparameter set optimized by the Bayesian method and that selected through experience.

Model	RMSE	MSE	MAE	*R* ^2^
GGRU	3.37	11.35	2.78	0.99
**BGGRU**	**2.66**	**7.11**	**1.99**	**0.99**

The bold part shows the best performance of the same indicator.

## Data Availability

Not applicable.
